# Recurrent Silent Thyroiditis as a Sequela of Postpartum Thyroiditis

**DOI:** 10.1155/2014/286373

**Published:** 2014-05-27

**Authors:** Preaw Hanseree, Vincent Bryan Salvador, Issac Sachmechi, Paul Kim

**Affiliations:** Department of Medicine, Icahn School of Medicine at Mount Sinai, Queens Hospital Center, Jamaica, NY 11432, USA

## Abstract

Thyroiditis encompasses a group of disorders characterized by thyroid inflammation. Though clinically indistinguishable from silent thyroiditis, postpartum thyroiditis occurs in women within 12 months after delivery. Recurrent postpartum thyroiditis in subsequent pregnancies is common, but recurrent silent thyroiditis is rare. We reported a case of patient with recurrent episodes of thyroiditis, unrelated to pregnancy, after an episode of postpartum thyroiditis. It is of interest that postpartum thyroiditis and silent thyroiditis could occur closely to each other; however, the link between these disorders is not well established. This report is to remind physicians of the possibility of recurrent silent thyroiditis in women with a history of postpartum thyroiditis.

## 1. Introduction

Painless or silent thyroiditis is a syndrome of thyrotoxicosis due to release of preformed thyroid hormones from disrupted thyroid follicles. Postpartum thyroiditis is indistinguishable from painless thyroiditis except that, by the definition, it occurs in women within 12 months after delivery [1, 2]. The prevalence of postpartum thyroiditis varies widely from 1.1 to 21.1% owing to the population studies and the frequency of monitoring [3]. Nikolai et al. [4] reported a prevalence of 6.7% in a North American population. After a first episode of postpartum thyroiditis, there is a 70% chance of recurrence with subsequent pregnancies [5]. While most of patients with painless thyroiditis have complete recovery and the recurrence is considered to be rare, we reported a case of patient with recurrent episodes of thyroiditis, unrelated to pregnancy, after an episode of postpartum thyroiditis.

## 2. Case Report

A 31-year-old pregnant woman previously diagnosed with hyperthyroidism was referred to thyroid clinic for comanagement of hyperthyroidism during pregnancy. She had been treated with propylthiouracil by her primary care physician on and off for the past 5 years; however the patient could not provide history of the exact nature of her thyroid condition. Initial thyroid functional panel was within normal limits. As patient was already in the second trimester, propylthiouracil was changed to methimazole until postpartum period. She remained euthyroid in the postpartum period and had negative thyroid stimulating immunoglobulin necessitating the discontinuation of methimazole. Scintigraphic thyroid imaging which was obtained postpartum and off methimazole demonstrated normal thyroid uptake (27.4%). Five months postpartum, she was diagnosed with postpartum thyroiditis manifesting with palpitations and tremors. There were no reported weight changes, eye changes, neck pain, chest pain, diaphoresis, diarrhea, constipation, heat or cold intolerance, and prior viral syndrome. She occasionally took naproxen and esomeprazole. Neck exam did not reveal neck tenderness or thyroid enlargement. Thyroid panel revealed suppressed TSH and elevated free T4 and T3. Thyroglobulin was elevated. Scintigraphic thyroid imaging was notable for decreased thyroid uptake at 0.6% (normal: 15–40%) (see Table 1). Symptomatic treatment was started with propranolol. After two months, she reverted back to euthyroid state which persisted for the next eleven months until she developed the first episode of recurrent thyroiditis. Thyroid panel during the recurrence was consistent with hyperthyroid state of thyroiditis. The second episode of recurrent thyroiditis occurred fourteen months after the previous episode. Thyroid panel (low TSH at 0.01 and elevated free T4 at 3.11) and scintigraphic thyroid scan (reduced thyroid uptake at 0.8%) were both consistent with hyperthyroid state of thyroiditis. She became euthyroid after 2 months. Further testing for thyroid antibodies revealed the presence of thyroglobulin antibody (49 IU/mL) and absence of TSH receptor blocking antibody, thyroid peroxidase antibody, and thyroid stimulating immunoglobulin. Heterophile antibody test repeatedly returned negative in 2 separate occasions. The patient remains euthyroid after the second episode of thyroiditis.

## 3. Discussion

There are several risk factors that may increase a woman's chance of developing postpartum thyroiditis. Women with the presence of thyroid peroxidase antibodies in early pregnancy have 30 to 50% chance of developing postpartum thyroiditis [6–9]. Moreover the titer of thyroid peroxidase antibodies is a predictor of the severity of postpartum thyroiditis and possible of recurrent disease [10, 11]. Women with a past history of thyroid disease have a 40% risk of developing postpartum thyroiditis, whereas those with type 1 diabetes mellitus or a family history of thyroid disease have a 20% risk [9]. Our patient had negative thyroid peroxidase antibodies, positive thyroglobulin antibodies, and past history of thyroid disease.

In women with positive thyroid peroxidase antibodies who recover from postpartum thyroiditis, there is a 70% recurrence rate in subsequent pregnancies [5–7]. The clinical features of silent thyroiditis are similar to postpartum thyroiditis; however, the recurrent rate is much lower. Nikolai et al. [12] reported recurrence rate of 10%, and Emerson and Farwell [13] reported less than 5%. To our knowledge, the largest number of recurrences noted in a single patient is nine [14].

Yamamoto et al. [15] have reported a patient with 7 episodes of silent thyroiditis within 4-year period, between 2 episodes of postpartum thyroiditis. Our patient had postpartum thyroiditis followed by 2 episodes of recurrent silent thyroiditis within 3-year period. Whether postpartum thyroiditis and silent thyroiditis are linked or interconnected is not clear. However, we understand that postpartum thyroiditis can be followed by episodes of silent thyroiditis based on our patient presentation, even though it is relatively uncommon.

Postpartum thyroiditis and silent thyroiditis are normally self-limited. Mild thyrotoxicosis rarely requires treatment, but when the disease is severe, it is symptomatically treated with beta-blockers. Antithyroid medication is not effective in preventing hormone release from the affected gland. Treatment of the hypothyroid phase may not be necessary, only if the patient is symptomatic or this phase is prolonged [1]. However, in patients with unusual repeated episodes of recurrent silent thyroiditis more definitive treatment may be warranted. Radioactive iodine therapy was reported to be effective in some cases of recurrent silent thyroiditis [14, 16–18]. Mittra and McDougall [14] reported four cases of recurrent silent thyroiditis wherein three of four patients who had 3, 4, and 9 episodes of thyrotoxicosis received radioablative iodine therapy with no further recurrence after the therapy; however, all of three patients became hypothyroid that required levothyroxine supplement. There have been reports of recurrent silent thyroiditis successfully treated with thyroidectomy as well [19]. We have discussed the options of radioablative iodine therapy and thyroidectomy with our patient to prevent further recurrence, but she declined at this time.

## 4. Conclusion

Although recurrent postpartum thyroiditis is common in subsequent pregnancies, recurrent silent thyroiditis unrelated to pregnancy following postpartum thyroiditis is rare. To our knowledge, we reported the second case of recurrent silent thyroiditis following postpartum thyroiditis. It is of interest that postpartum thyroiditis and silent thyroiditis could occur closely to each other. This report is to remind physicians of the possibility of recurrent silent thyroiditis in women with history of postpartum thyroiditis.

## Figures and Tables

**Table 1:** Temporal profile of thyroid function test and thyroid uptake scan for the patient reported to be having recurrent thyroiditis.

Date	Free T4 (0.58–1.64 ng/dL)	T3 (87–178 ng/dL)	TSH (0.35–3.5 mIU/mL)	24-h RAIU (15–40%)	Diagnosis
November 2010 (1 month postpartum)	0.76	157	1.05	27.4%	Euthyroid
March 2011 (5 months postpartum)	2.09 ↑	212.6 ↑	0.00 ↓	0.6% ↓	**Postpartum thyroiditis**
December 2011			2.98		Euthyroid
May 2012 (19 months postpartum)	3.10 ↑	269.8 ↑	0.02 ↓		**1st recurrent thyroiditis**
November 2012	0.93		2.97		Euthyroid
February 2013	0.78	70.28 ↓	4.79 ↑		Hypothyroid
July 2013 (33 months postpartum)	3.11 ↑		0.01 ↓	0.8% ↓	**2nd recurrent thyroiditis**
September 2013	0.60		0.81		Euthyroid
